# Augmented serum level of major histocompatibility complex class I-related chain A (MICA) protein and reduced NKG2D expression on NK and T cells in patients with cervical cancer and precursor lesions

**DOI:** 10.1186/1471-2407-8-16

**Published:** 2008-01-21

**Authors:** Naela A Arreygue-Garcia, Adrian Daneri-Navarro, Alicia del Toro-Arreola, Angel Cid-Arregui, Oscar Gonzalez-Ramella, Luis F Jave-Suarez, Adriana Aguilar-Lemarroy, Rogelio Troyo-Sanroman, Alejandro Bravo-Cuellar, Vidal Delgado-Rizo, Trinidad Garcia-Iglesias, Georgina Hernandez-Flores, Susana del Toro-Arreola

**Affiliations:** 1 Laboratorio de Inmunología, Departamento de Fisiología, Centro Universitario de Ciencias de la Salud, Universidad de Guadalajara, Guadalajara, Jalisco, México; 2 Tumor Gene Therapy German Cancer Research Center (DKFZ) Im Neuenheimer Feld 280 69120-Heidelberg, Germany; 3 División de Inmunología, Centro de Investigación Biomédica de Occidente, Instituto Mexicano del Seguro Social, Guadalajara, Jalisco, México

## Abstract

**Background:**

Cervical cancer is the second most common cancer in women worldwide. NK and cytotoxic T cells play an important role in the elimination of virus-infected and tumor cells through NKG2D activating receptors, which can promote the lysis of target cells by binding to the major histocompatibility complex class I-related chain A (MICA) proteins. Increased serum levels of MICA have been found in patients with epithelial tumors. The aim of this study was to compare the levels of soluble MICA (sMICA) and NKG2D-expressing NK and T cells in blood samples from patients with cervical cancer or precursor lesions with those from healthy donors.

**Methods:**

Peripheral blood with or without heparin was collected to obtain mononuclear cells or sera, respectively. Serum sMICA levels were measured by ELISA and NKG2D-expressing immune cells were analyzed by flow cytometry. Also, a correlation analysis was performed to associate sMICA levels with either NKG2D expression or with the stage of the lesion.

**Results:**

Significant amounts of sMICA were detected in sera from nearly all patients. We found a decrease in the number of NKG2D-expressing NK and T cells in both cervical cancer and lesion groups when compared to healthy donors. Pearson analysis showed a negative correlation between sMICA and NKG2D-expressing T cells; however, we did not find a significant correlation when the analysis was applied to sMICA and NKG2D expression on NK cells.

**Conclusion:**

Our results show for the first time that high sMICA levels are found in sera from patients with both cervical cancer and precursor lesions when compared with healthy donors. We also observed a diminution in the number of NKG2D-expressing NK and T cells in the patient samples; however, a significant negative correlation between sMICA and NKG2D expression was only seen in T cells.

## Background

Cervical cancer is the second most common malignant tumor in women worldwide, and the most common tumor in developing countries including Mexico, the site of this study [1-3]. Infection with high-risk human papilloma virus (HPV) is considered the major etiological factor of HPV-related premalignant lesions and cervical cancer [4-7]. Virtually all cervical carcinoma patients (99.7%) have shown to be HPV-DNA carriers [8]. Although HPV prevalence is very common in sexually active women [9], the infection in the majority of cases is transient, clearing in a short period of time without progression to clinical lesions [10-12]. In a minority of cases, HPV presence is established as a persistent infection. It is thought that viral persistence leads to progression from low-grade squamous intraepithelial lesions (LSIL) to high-grade squamous intraepithelial lesions (HSIL) and eventually to invasive carcinoma [13-16]. The progression of the lesions may involve an adverse tumor environment, wherein the mucosal immune response may be unable to eradicate malignant cells.

The innate immune response is considered to be the first line of defense at mucosal surfaces. NK cells are an important arm of the innate immune system specialized for killing virus-infected and tumor cells [17]. The activity of NK cells is tightly regulated by a complex balance of inhibitory and activating receptors [18]. NKG2D is a C-type lectin-like activating receptor encoded within the NK gene complex on human chromosome 12 [19]. NKG2D is expressed in almost all NK cells and a variety of T cell subsets such as CD8^+ ^T cells [20]. NKG2D can promote tumor lysis by binding to a recently identified family of cell surface ligands encoded by the MHC class I chain-related (MIC) genes [21-23]. MIC proteins are a novel family of non-classical MHC class I molecules. The MIC family includes MICA, which is a highly polymorphic functional cell-surface protein [24]. Similar to classical HLA class I molecules, MICA also contains three extracellular domains (α1-α3); however, MICA neither associates with β_2_-microglobulin nor binds antigen peptides. Under normal physiological conditions, MICA expression is almost restricted to the gastrointestinal epithelium [25,26]; nevertheless, MICA is overexpressed in several epithelial tumors [27-30]. This finding has led to the proposal that MICA is a stress cell marker in nascent epithelial tumors [26].

Available evidence suggests that NKG2D engagement by MICA induces proliferation, survival, and, cytotoxic activity in NK cells [31,32]. Consequently, NKG2D/MICA interaction may represent an important activation pathway to trigger the immune attack against tumor cells [30,33]. However, it has been demonstrated that MICA shedding from tumor cell surface occurs in a variety of malignant epithelial tumors, including advanced hepatocellular carcinoma, colon, prostate, renal, and breast cancer, as well as hematopoietic tumors [34-38]. Accumulation of sMICA in serum may lead to the down-modulation of NKG2D through the facilitation of NKG2D internalization and lysosomal degradation. This strategy has been proposed to be a novel mechanism used by cancer cells to evade the tumor immunosurveillance [39].

In this study, we formulated the hypothesis that sMICA levels increase concomitantly with the natural history of cervical cancer in the progression from intraepithelial precursor lesion to invasive cancer. To address this assertion, we compared the sMICA levels and the number of NKG2D-expressing NK and T cells in blood samples from patients with cervical cancer or precursor lesions with those from healthy donors. We also performed a correlation analysis to explore a relationship between sMICA levels and NKG2D expression. Furthermore, we associated sMICA levels with the stage of the lesion.

## Methods

### Patients

In accordance with the FIGO (International Federation of Gynecology and Obstetrics) System of Clinical Staging [40,41], we included 10 patients with invasive squamous cell carcinoma of the uterine cervix and 17 patients with squamous intraepithelial lesion, which were classified as high-grade squamous intraepithelial lesion (HSIL) or low-grade squamous intraepithelial lesion (LSIL) following the 2001 Bethesda System for reporting cervical or vaginal cytologic diagnoses [42,43]. All patients enrolled in the study were first subjected to colposcopic evaluation. Ten age/gender matched healthy donors were also included as control group. All patients were attended at the Oncology and Gynecology Department, Hospital Civil de Guadalajara, Mexico.

Peripheral blood (PB) samples were taken after written informed consent was obtained from all patients (LSIL, HSIL, and cervical cancer) and healthy donors. Samples were collected in heparinized tubes to separate peripheral blood mononuclear cells (PBMC). PB was also obtained without heparin in order to separate serum, which was stored at -20°C until use.

### Ethical considerations

The present study was approved by the Research Ethics Committee in Biomedical Sciences (Universidad de Guadalajara, Guadalajara, Jalisco, Mexico and Hospital Civil de Guadalajara, OPD, Guadalajara, Jalisco, Mexico. Trial reference: CSIM 200-22, 20000302032), in accordance with the guidelines of the Mexican Official Standard (Norma Oficial Mexicana NOM) and the World Medical Association Declaration of Helsinki (adopted by the 52^nd ^WMA General Assembly, Edinburgh, Scotland, October 2000).

### Quantification of sMICA in serum

MICA ELISA kit (Immatics Biotechnologies, Tübingen, Germany) was used to detect sMICA in serum samples, according with the manufacturer protocol. Briefly, after covering the 96 well flat-bottom plates with capture anti-MICA mAb, standard serial dilutions and serum samples were added to each well. Detection anti-MICA mAb was added to the wells. HRP-conjugated anti-mouse Ab (Southern Biotechnologies, Birmingham, AL, USA) was added and color was developed using tetramethylbenzidine system (KPL, Gaithersburg, MD, USA). Absorbance values (at A_450_) by duplicate were plotted against dilutions and expressed as pg/mL (normalized to log_10_).

### NKG2D expression on NK and T cells

Surface NKG2D expression was evaluated by flow cytometry. Briefly, PBMC were obtained by Ficoll-Hypaque density gradient centrifugation. After isolation, PBMC were adjusted at 8 × 10^5 ^cells/mL and incubated with mouse anti-NKG2D primary mAb (kindly donated by Professor Alessandro Moretta, University of Genova, Italy) for 30 min at 4°C. Cells were washed with PBS and incubated with goat anti-mouse IgG FITC-conjugate as a secondary reagent for 30 min at 4°C in the dark. Afterward, cells were washed and incubated with PE-conjugated anti-CD56, and PC5-conjugated anti-CD3 mAb; matching isotype controls were also included. Finally, cells were fixed with 0.5% paraformaldehyde solution. A three-color analysis on an EPICS XL-MCL Flow Cytometer (Beckman Coulter, Krefeld, Germany) was used to determine the NKG2D expression on CD56^+^CD3^- ^and CD56^-^CD3^+ ^populations.

### Statistical analysis

Statistical analysis of all data was done using SPSS software version 10 (Chicago, IL, USA). The sMICA levels and percentages of NKG2D-expressing NK and T cells were expressed as mean ± SD. Statistical comparisons among different groups were performed using a non-parametric test (Mann-Whitney test). Pearson analysis was performed to correlate sMICA levels with NKG2D expression and Spearman analysis was performed to correlate sMICA levels with the stage of the lesion. Additionally, a multivariate regression analysis was achieved to evaluate whether sMICA and NKG2D expression act in concert during the cervical cancer progression. For this analysis we used the method "forward stepwise" in which we added the independent variables (sMICA and NKG2D expression) one by one. A 95% confidence interval (*p *< 0.05) was considered statistically significant.

## Results

Ten patients with established histopathological diagnosis of uterine cervix invasive squamous cell carcinoma were enrolled in this study. Additionally, a group of 17 patients with squamous intraepithelial lesions was composed of 7 individuals diagnosed as high-grade and 10 individuals diagnosed as low grade. We also included a control group matched in age/gender as shown in Table 1. Clinical and laboratory parameters confirmed that patients and controls did not have any autoimmune or blood disorder that could alter our study variables (sMICA level and NKG2D expression).

**Table 1:** Patient characteristics

***Study group***		***n***	***Age (years)***

Healthy donors		10	27–52 *Median age = 38.4*
SIL	LSIL	10	26–50 *Median age = 36.6*
	HSIL	7	23–49 *Median age = 35.0*
Carcinoma		10	29–69 *Median age = 45.4*

### sMICA levels are preferentially augmented in patients with cervical cancer and precursor lesions

**Figure 1:**
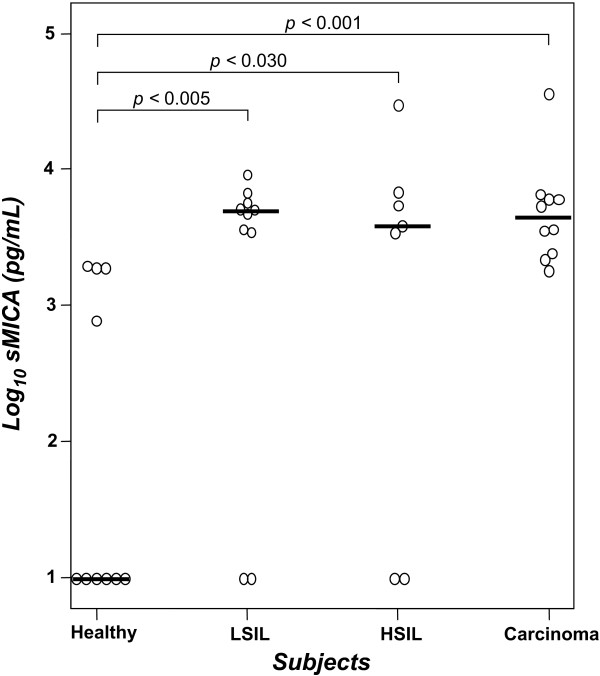
Serum sMICA is elevated in cervical cancer patients. sMICA was determined by using ELISA assay. Short horizontal lines indicate the median value (pg/mL normalized to log_10_) in each group. Statistical analysis among all groups was performed by Mann-Whitney test.

We investigated the sMICA level in sera from cervical carcinoma patients and SIL patients (including both high-grade and low-grade lesions). Additionally, we also tested sMICA in healthy individuals. In order to normalize the sMICA values, we converted the data (expressed as pg/mL) to log_10_. As shown in Figure 1, we found significantly higher sMICA levels in patients with cervical cancer or precursor lesions when compared with healthy donors (Mann-Whitney test, *p *< 0.005 in LSIL, *p *< 0.030 in HSIL, and *p *< 0.001 in cancer group). Interestingly, the majority of healthy individuals showed almost undetectable sMICA level (lower than 1 pg/mL). In contrast, measurable sMICA level was found in patients with cervical cancer and precursor lesions. The highest sMICA level was found in the cancer group (4.56 pg/mL). We also examined a potential relationship between sMICA level and the stage of the lesion. Spearman correlation showed that sMICA level was significantly positively correlated with the severity of the lesion (Rho = 0.519; *p *< 0.001).

### Reduced numbers of NKG2D-expressing NK and T cells in patients with cervical cancer and precursor lesions

**Figure 2:**
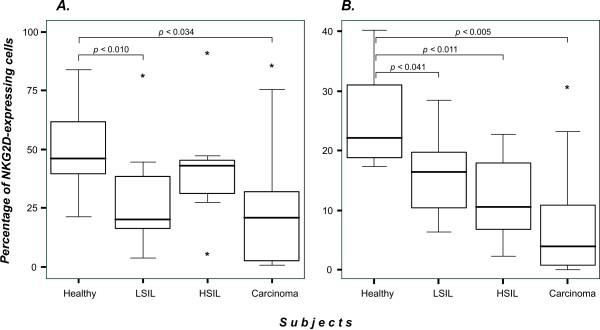
NKG2D-expressing immune cells are decreased in patients with cervical cancer and precursor lesions. Three color flow cytometry analysis to detect CD3, CD56 and NKG2D was carried out on PBMC to determine the percentage of NKG2D-positive cells. ***A) ***NKG2D-expressing NK cells (CD56^+^CD3^- ^population); ***B) ***NKG2D-expressing T cells (CD56^-^CD3^+ ^population). The box plots represent each study group. Medians are represented as thick horizontal lines, 25^th ^and 75^th ^percentiles as boxes and 10^th ^and 90^th ^percentiles as whiskers. *Extreme values.

NKG2D plays an important role in the immune recognition of tumor targets after engagement by MICA molecules [31,32]. Nevertheless, there is evidence showing that circulating sMICA interferes with NKG2D expression on NK and T cells [39]. Owing to the observed increase in the sMICA levels detected in sera from patients with cervical cancer and those with precursor lesions, we investigated expression of the NKG2D receptor on peripheral NK and T cells. First, we evaluated the percentage of NK and T cells in all patients and healthy donors. The results fell into the normal range in all groups, with a non-significant drop in cancer group (data not shown). In order to determine NKG2D expression on NK cells, we gated the CD56^+^CD3^- ^population. As shown in Figure 2A and Table 2, we found the following results: 50.40% of the gated population was NKG2D-positive in the control group, 28.57% in LSIL, 40.90% in HSIL and 27.49% in cancer group. Statistical comparison between the cancer and control groups revealed a significant difference (*p *< 0.034).

**Table 2:** NKG2D-expressing NK and T cells

	**Percentage of NKG2D-expressing cells**							
	
**Subjects**	***CD56*^+^*CD3*^-^**				***CD56*^-^*CD3*^+^**			
	
	*Mean* *± SD*	*Min*	*Max*	*p*	*Mean* * ± SD*	*Min*	*Max*	*p*
Healthy	50.40 ± 20.78	21.44	83.84		24.80 ± 7.67	17.30	40.20	
LSIL	28.57 ± 1.36	3.60	78.40	0.010	15.74 ± 6.91	6.40	28.50	0.041
HSIL	40.90 ± 5.67	2.70	88.10	0.495	12.20 ± 7.49	2.40	22.80	0.011
Invasive carcinoma	27.49 ± 9.62	0.50	82.90	0.034	8.28 ± 0.25	0.10	29.60	0.005

*p *represents the value obtained of comparing LSIL, HSIL, and carcinoma subjects *vs *healthy subjects

We also analyzed the CD56^-^CD3^+ ^population to determine NKG2D expression in T cells. We observed a decrease in the number of NKG2D-expressing T cells concordant with the severity of the lesion, as can be observed in Figure 2B and Table 2. The control group showed 24.80% of the gated population to be NKG2D-positive cells. In contrast, LSIL, HSIL, and cancer groups showed NKG2D positive percentages of 15.74, 12.20, and 8.28, respectively. Statistical analysis revealed a significant difference in both precursor lesion and cancer groups when compared to healthy individuals (*p *< 0.041, *p *< 0.011, and *p *< 0.005 to LSIL, HSIL, and cancer groups, respectively).

### Circulating sMICA can reduce the number of NKG2D-expressing T cells

**Figure 3:**
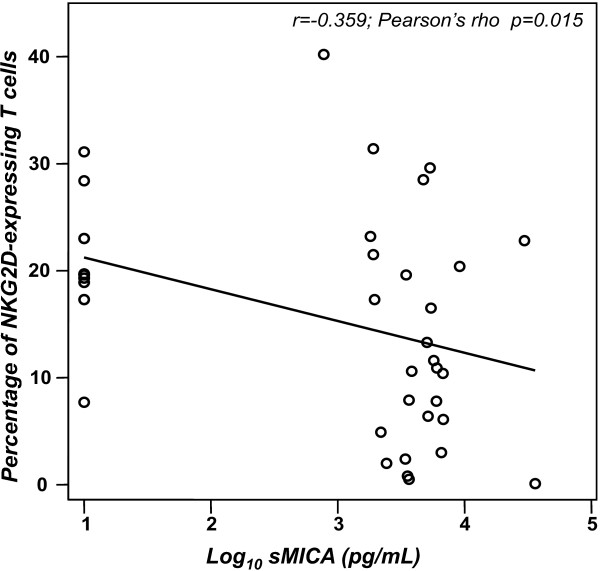
sMICA levels and the number of NKG2D-expressing T cells are negatively correlated. Correlation analysis was done using the Pearson test. Values are normalized as log_10_.

**Figure 4:**
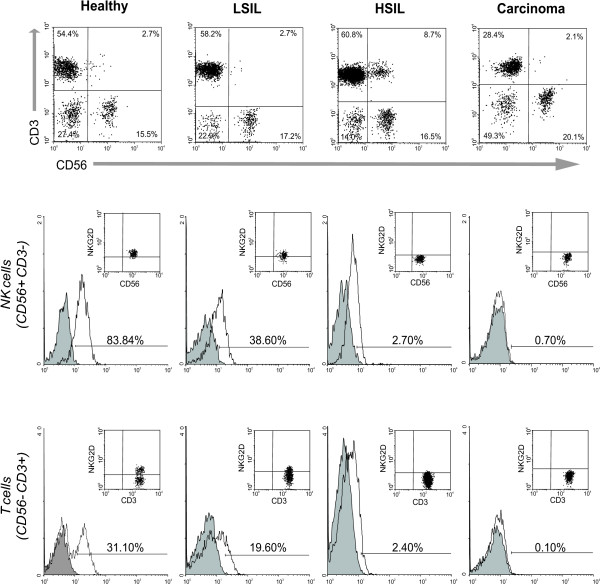
The number of NKG2D-expressing NK and T cells diminishes during cervical cancer progression. NKG2D expression was measured using flow cytometry while gating on CD56^+^CD3^- ^(NK cell) and CD56^-^CD3^+ ^(T cell) populations. The histograms and dot plots show the results obtained from a representative individual of each group. The same tendency is observed in both NK and T cells (filled curve: isotype control Ab, open curve: anti-NKG2D).

It has been reported that sMICA in serum from patients with cancer induces down-modulation of surface NKG2D expression [39]. For this reason, we investigated a potential correlation between sMICA levels and the percentage of NKG2D-expressing NK and T cells. As shown in Figure 3, sMICA levels were negatively correlated with the number of NKG2D-expressing T cells (r = -0.359; *p *< 0.015). In contrast, we did not observe any significant correlation between sMICA level and NKG2D-expressing NK cells (data not shown). It is important to mention that despite the lack of statistical correlation, we observed a substantial decrease of NKG2D-expressing NK cells in more than a half of the analyzed patients with cervical cancer. Figure 4 shows representative histograms and dot plots of NKG2D-expressing NK and T cells in all groups.

We used a multivariate regression analysis to determine whether increasing sMICA levels and decreasing NKG2D expression on T cells could act in concert to predict the progression of the lesion advancing toward cancer. The results derived from that analysis (Table 3) show that both categories (sMICA level and NKG2D) were significantly strongly related with the progression of the lesion (R^2 ^= 0.467; *p *< 0.001). The importance of these results is that R^2 ^explains (46.7%) of the variation in the progression toward cancer.

**Table 3:** Multivariate regression analysis of sMICA and NKG2D-expressing T cells acting in concert in the progression of the lesion

	***Regression coefficients***		***Standardized coefficient***			***95% C.I. for B***	
				
	***B***	***SEM for B***	***Beta***	***t***	***Sig***.	***Lower limit***	***Upper limit***
**Constant**	2.417	0.532		4.544	0.000	1.336	3.497
**Log**_10_**sMICA**	0.314	0.128	0.330	2.460	0.019	0.055	0.574
**% NKG2D-expressing T cells**	-0.057	0.015	-0.492	-3.667	0.001	-0.088	-0.025

**B: **regression coefficient in the equation which predicts the grade of the lesion considering to both sMICA and NKG2D expression as independent variables; **SEM for B: **standard error for the regression coefficients; **Beta: **standardized regression coefficient which determines the actual weight of the regression coefficient if all variables are expressed in standard units; **t: **t-test value for the regression coefficient; **sig: ***p *value **(R^2 ^= 0.467; *p *< 0.001)**.

## Discussion

Recent reports have revealed a new tumor evasion strategy involving MICA release from malignant cell surface in different human tumors [34-37]. In this study we found significant sMICA levels in patients with cervical cancer. Proteolytic shedding has been proposed as the key mechanism by which MICA is released from the cell surface, similar to the cleavage occurring with other membrane-bound proteins. By using different protease inhibitors, it was elucidated that metalloproteinases were responsible for the cleavage of the MICA α1α2α3 extracellular domain from the cell surface in several tumor lines [44]. This finding may have implications to cervical cancer as previous data in our laboratory have shown extensive metalloproteinase activity in cervical tissue extracts from patients with cervical cancer and precursor lesions [45].

This suggests that metalloproteinases, aside from playing an important role in invasion, metastasis, and angiogenesis processes, may also have a role in allowing MICA shedding tumor cells to evade immune attack. However, we cannot discard the possibility that other different proteases are also participating in the cleavage of MICA as has been recently reported by Kaiser *et al*. In that report, the authors demonstrated that MICA shedding was facilitated by a disufide-isomerase interacting directly with MICA α3 [46]. Thus, it appears that different enzymes could be enabling tumor cells to escape from the immune attack. Since in this study we did not examine the relevance of the proteolytic activity, it will be of particular importance to characterize the enzymatic activity with respect to cell surface MICA shedding in cervical cancer.

We observed higher levels of sMICA in patients with low-grade intraepithelial lesions in comparison with healthy individuals; these levels increased in accordance with cancer progression. This finding is in agreement with recent results obtained by Wu *et al*., who detected elevated amounts of sMICA in patients with prostate cancer [36]. Moreover, significantly higher levels were seen in patients with more advanced diseases, suggesting that the MICA shedding may contribute to prostate cancer progression Thus, consistent with Wu *et al*., we could speculate that high levels of circulating sMICA that we observed may be contributing to the immune tolerance observed in patients with cervical cancer. However, functional experiments to elucidate the actual relevance of MICA/NKG2D pathway in cervical cancer will be necessary.

Different studies have proven the down-modulation of HLA class I expression during cervical cancer progression [47,48]. Hence, it is feasible to assume that NK cells may represent an important immune defense against cervical cancer. Different activating receptors confer to NK cells the capacity to kill virus-infected or tumor cells [49,50]; one of these receptors is represented by NKG2D, which can recognize different ligands including MICA molecules; thus, NKG2D could have a significant role in anti-tumor immune response [21-23].

Recently, Doubrovina *et al*., have established that MICA in serum from patients with colon adenocarcinoma down-modulates NKG2D expression on NK cells via its internalization and subsequent lysosomal degradation [35]. Importantly, we observed a reduction of NKG2D-expressing NK cells in patients with cervical cancer and precursor lesions; however, we did not find a significant statistical correlation between sMICA and lower NKG2D expression on these cells. These results indicate that additional factors could be also affecting the surface NKG2D expression. Lee *et al*., have provided evidence showing that TGF-β1 present in plasma of lung and colorectal cancer patients impairs NK cell activity via NKG2D down-modulation [51]. Using a technology to silence TGF-β1 and -β2 genes in malignant glioma cells, Friese *et al*. revealed that these tumor cells did not down-modulate the NKG2D expression on NKL cells (a NK cell line); moreover, strong surface MICA expression on tumor cells was also observed when they blocked the TGF-β production promoting a strong recognition by immune cells [52]; these results support the key role of TGF-β in the MICA/NKG2D pathway. It is well known that this cytokine is largely produced by many tumor cells and it is also common in cervical squamous intraepithelial lesions [53]. For instance, it has been shown that HPV-11 transformed human tissue over-expresses TGF-β1 [54] and benign cervical lesions, particularly, have been associated with HPV-6 or -11.

It has been recently observed that NKp44, another important receptor confined only to activated NK cells, becomes down-modulated on NK cells from both healthy donors and patients with cancer upon exposure to sMICA-containing serum [35]. Taking into account that both NKG2D and NKp44 on the cell surface may be down-modulated by sMICA, it is viable to consider that circulating sMICA could be affecting the integration of different crucial triggering signals in NK cells. Collectively, these alterations may be contributing to the incomplete NK cell-mediated cytotoxic function in patients with cancer.

NKG2D expression is not only confined to NK cells, but it is also expressed on CD8^+ ^cytotoxic T cells, where this receptor confers co-stimulatory signals [55]. Moreover, NKG2D expression has also been described in a subset of CD4^+ ^T cells from patients with some inflammatory diseases [56]. For this reason, we also evaluated surface NKG2D expression on T cells and analyzed its correlation with sMICA level. Despite finding a statistically significant correlation between sMICA level and NKG2D-expression, our analysis only showed a weak correlation, which suggests the existence of additional factors that, in concert with sMICA, could promote the NKG2D down-modulation; one of such factors could be TGF-β1 as previously mentioned. In contrast to the several reports supporting the role of sMICA in the NKG2D down-modulation [39], there is a recent report by Osaki *et al*., which showed that sMICA levels were not different between gastric cancer patients and normal controls, indicating that sMICA was not responsible for inducing the NKG2D down-modulation on CD8^+ ^T cells from those patients. However, transwell experiments demonstrated that direct contact between CD8^+ ^T cells and MICA-expressing tumor cells caused NKG2D down-modulation, indicating that surface MICA was responsible for that defect [57]. Independent of its form (sMICA or membrane MICA), it is clear that this molecule promotes, in part at least, the NKG2D down-modulation in immune cells. Since in this study we only used an anti-CD3 antibody, we were not able to distinguish between NKG2D-expressing CD8^+ ^or CD4^+ ^T cells; thus, this methodological flaw could also explain the weak correlation seen between sMICA level and NKG2D-expressing T cells. Therefore, the analysis to distinguish CD8^+ ^or CD4^+ ^T cells would have been more interesting and meaningful that our present results, which were confined to the total T cell population. Future studies are planned to identify the actual T cell population in which cell surface NKG2D is being affected. Consequently, it will be possible to discern if specific T cell lineages fail to be fully activated in patients with HPV-associated cervical lesions.

In this study we have demonstrated a reduction in the number of NKG2D-expressing immune cells. At the moment, we do not know the meaning of this finding; however, we consider that this reduction was not because a total lack of surface NKG2D, due to the flow cytometry analysis did not reveal the presence of more than a peak in the histograms (Figure 4). We cannot discard the possibility that the reduction in the number of NKG2D-expressing cells could be attributed to cell death. In this study we did not examine apoptosis in NK or T cells, and we also did not look for sMICA bound to the NKG2D receptor; therefore, we cannot know if a sMICA-induced apoptosis mechanism in immune cells exists in patients with cervical cancer and precursor lesions. The above phenomenon has been reported with non-classical HLA molecules, such as HLA-G, which has multiple immunoregulatory properties, mainly exemplified in the abrogation of maternal NK cell activity [58]. This molecule is expressed not only as a cell surface molecule, but also as a soluble form which has been found in sera from patients undergoing different malignancies [59-61]. Interestingly, soluble isoforms of HLA-G have shown to trigger *in vitro *apoptosis in both activated CD8^+ ^cytotoxic NK and T cells through a Fas/FasL mechanism [62,63]. In consequence, this immunoregulatory role used by secreted forms of HLA-G could represent an additional mechanism by which tumors avoid the immune surveillance. Therefore, it will be interesting to investigate if soluble HLA-G is present in cervical cancer patients and especially, to examine if serum HLA-G and MICA altogether are able to deliver a death signal in immune cells.

Finally, the results derived from the multivariate regression analysis statistically show that an increase in sMICA level may be associated with progression to invasive cancer; while an increase of NKG2D-expressing T cells could be negatively correlated with cancer progression. We did not find any significant correlation when we extended the same analysis to NKG2D-expressing NK cells. Thus, it will be interesting to design functional experiments to test whether T cells are being affected by sMICA, even more, it will be necessary to study separately CD4^+ ^and CD8^+ ^T cells to discern their actual correlation with sMICA.

## Conclusion

Our study reveals for the first time that sMICA is elevated in patients with cervical cancer and precursor lesions. We also demonstrated a reduced number of NKG2D-expressing NK and T cells in patient samples. Furthermore, a significant correlation between sMICA and NKG2D expression was found in T cells, but not in NK cells. If confirmed that sMICA is directly contributing to the immune evasion present in patients with cancer, targeting specific proteases involved in MICA shedding from cell surfaces or directly blocking sMICA in circulation could be a clinically important strategy to boost the anti-tumor response against cervical cancer.

## Competing interests

The author(s) declare that they have no competing interests.

## Authors' contributions

NAG performed the experimental work described in the study, searched scientific literature, contributed with the draft and edited the manuscript; ADN contributed to the planning of the project; participated in its coordination and provided valuable scientific suggestions; ATA was the core in the flow cytometry experiments and performed research; ACA participated in the design of the study and contributed to the review of the manuscript; OGR contributed to the draft of the manuscript and helped with editing; LJS, and AAL oversaw the experiments, contributed with scientific ideas and assisted with the writing; RTS supported with the statistical analysis of the data; VDR, and TGI helped in the ELISA experiments; ABC and GHF contributed with scientific ideas and research; STA conceived and designed the theoretical framework of the study, provided scientific guidance throughout the project and wrote the manuscript. All authors read and approved the final manuscript.

## Pre-publication history

The pre-publication history for this paper can be accessed here:


